# Physician-Investigator, Research Coordinator, and Patient Perspectives on Dual-Role Consent in Oncology: A Qualitative Study

**DOI:** 10.1001/jamanetworkopen.2023.25477

**Published:** 2023-07-25

**Authors:** Stephanie R. Morain, Dorit Barlevy, Steven Joffe, Emily A. Largent

**Affiliations:** 1 Johns Hopkins Berman Institute of Bioethics and the Department of Health Policy and Management, Johns Hopkins Bloomberg School of Public Health, Baltimore, Maryland; 2 Center for Medical Ethics and Health Policy, Baylor College of Medicine, Houston, Texas; 3 Department of Medical Ethics and Health Policy, University of Pennsylvania Perelman School of Medicine, Philadelphia; 4 Leonard Davis Institute of Health Economics, University of Pennsylvania, Philadelphia

## Abstract

**Importance:**

Classic statements of research ethics generally advise against dual-role consent in which physician-investigators seek consent for research participation from patients with whom they have preexisting treatment relationships. Yet dual-role consent is common in clinical oncology research, as studies are often conducted in close relationship with clinical care.

**Objective:**

To explore key stakeholders’ perspectives on dual-role consent in clinical oncology trials.

**Design, Setting, and Participants:**

This qualitative study with 43 participants was conducted at a National Cancer Institute–designated comprehensive cancer center from 2018 to 2022. Semistructured qualitative interviews of physician-investigators, research coordinators, and patients were performed. Respondents were recruited from 3 populations: (1) physician-investigators engaged in clinical oncology research; (2) research coordinators engaged in clinical oncology research; and (3) patients, with and without prior clinical trial experience, who had received a new cancer diagnosis at least 2 months prior to enrollment in this study.

**Main Outcomes and Measures:**

Interviews were audio recorded and professionally transcribed. A thematic analysis approach was used to develop a codebook that included both theory-driven, a priori codes and emergent, inductive codes. Two authors double-coded all transcripts and met regularly to compare coding, discuss discrepancies, refine the codebook, and draft memos describing relevant themes and their frequency.

**Results:**

Among the 43 respondents, 28 (65.1%) were female; 9 (20.9%) were African American, 8 (18.6%) were Asian, 6 (14.0%) were Hispanic, and 21 (48.8%) were White; 15 were physician-investigators (6 [40.0%] with 6-10 years of experience, 4 [26.7%] with at least 20 years of experience), 13 were research coordinators (5 [38.5%] with 0-5 years of experience, 5 [38.5%] with 6-10 years of experience), and 15 were patients (9 [60.0%] aged 46-64 years). Four main themes were found: interviewees (1) perceived greater potential for role synergy than for role conflict; (2) reported dual-role consent as having mixed effects on the consent process, increasing prospective participants’ understanding and likelihood of agreement while also challenging voluntariness; (3) preferred a team-based approach to the consent process in which physician-investigators and research coordinators share responsibility for communicating with prospective participants and safeguarding voluntariness; and (4) offered strategies for managing tensions in dual-role consent.

**Conclusions and Relevance:**

This qualitative study found that concerns about dual-role consent in clinical oncology, while valid, may be outweighed by corresponding advantages, particularly if appropriate mitigation strategies are in place. These findings support a team-based approach to informed consent, in which physician-investigators and research coordinators promote both the understanding and voluntariness of prospective participants.

## Key Points

**Question:**

How does involvement of physician-investigators in seeking consent for clinical oncology trials from prospective participants with whom they have a preexisting treatment relationship (ie, dual-role consent) advance or impede the goals of informed consent?

**Findings:**

In this qualitative study, respondents perceived the benefits of dual-role consent for clinical oncology research, including supporting understanding and cohering with participant preferences, as outweighing potential risks, including compromising voluntariness and exacerbating therapeutic misconception.

**Meaning:**

These findings suggest support for a team-based approach to informed consent for oncology research, in which physician-investigators and research coordinators collaborate to meet prospective participants’ needs.

## Teaser

This qualitative study investigates key stakeholders’ perspectives on dual-role consent in clinical oncology trials.

## Introduction

Informed consent is considered an ethical requirement of most research because it secures prospective participants’ rights to make autonomous decisions about research participation, demonstrating respect for them as persons.^1^ Furthermore, there is consensus that the consent process begins with the initial approach to the prospective participant, includes the sharing of key information about the study, and culminates in a voluntary decision about participation.

Controversy exists, however, around who can permissibly seek informed consent. What role, if any, can a prospective participant’s treating physician play in the consent process when that physician leads or collaborates in said research?^2,3,4,5,6,7,8^ Previously, we have used the term dual-role consent to describe the practice of physician-investigators seeking consent for research participation from individuals with whom they have preexisting treatment relationships.^2^

Classic statements of research ethics advise against dual-role consent, based on the view that distinct normative commitments govern physician-patient and investigator-participant relationships.^9,10^ For instance, the American Medical Association advises that “someone other than the treating physician [should obtain] the participant’s informed consent.”^11^ Although federal research regulations do not address dual-role consent, the Office for Human Research Protections, which provides federal leadership on human research in the United States, cautions against dual-role consent in guidance documents, saying “patients might feel obligated to participate in research if their physician is also the investigator.”^12^

Three worries underlie this stance.^2^ First, simultaneously wearing the hats of both physician and investigator can result in role conflict (tension between the duties of care and of research) for the physician-investigator. Second, dual-role consent might reduce the voluntariness of consent if the patient-participant feels unable or unwilling to decline. Third, dual-role consent might promote therapeutic misconception, or confusion of the goals of research with the goals of care, in the patient-participant.

Dual-role consent is common in clinical oncology research.^13,14,15,16,17^ This mismatch between guidance and practice suggests a need either to (1) reform practice to cohere with normative guidance (ie, refrain from dual-role consent) or (2) revise norms to accord with practice (ie, endorse dual-role consent).^2^ A third way might incorporate reform and revision (eg, specify when dual-role consent is and is not permissible and change practice accordingly). Understanding which of these options is preferable requires empirical insights.^18^

Existing data on dual-role consent are limited but suggest oncologists hold mixed views.^12,13,14^ Studies of recruitment for pediatric oncology trials suggest some physician-investigators do not recruit their own patients, whom they see as “vulnerable [and] in need of protection.”^19^ These same clinicians worry about the impact of research participation on the physician-patient relationship.^19^ Other clinicians suggest dual-role consent might facilitate communication and decision-making; from their perspective, the physician-investigator may have unique insights into the patient’s particular medical situation, values, and goals of care.^19,20,21^ Limited data suggest investigator involvement may be associated with increased understanding among participants.^22^ Notably, these prior studies have generally focused on pediatric populations, which differ in important ways from adults.^23^ Additionally, these studies have been conducted in the Netherlands and the United Kingdom; differences in how the US organizes and finances both care and research may yield different results.

Empirical evidence suggests patients may prefer to discuss research participation with their treating physicians, though this is derived primarily from comparative effectiveness research contexts.^24,25,26^ Furthermore, the perspectives of noninvestigator study staff members (eg, research coordinators and research nurses) have generally been overlooked, despite these individuals often having considerable frontline research experience, including in consent processes.^27^

To advance understanding of dual-role consent for adult oncology research, we interviewed physician-investigators, patients, and research coordinators. This research leverages qualitative methods to understand the challenges and advantages of dual-role consent from key stakeholders’ perspectives.

## Methods

This study followed the Standards for Reporting Qualitative Research (SRQR) reporting guideline.^28^ It was approved by the Baylor College of Medicine and Johns Hopkins Bloomberg School of Public Health institutional review boards. Interviewees gave verbal consent. Proportionate to their expected interview length, patients and research coordinators received $20 and $40, respectively; physician-investigators received no compensation. The research team was composed of doctoral-level researchers with diverse disciplinary backgrounds, including medical and research ethics, health policy, law, nursing, and pediatric oncology.

### Sampling and Recruitment

All interviewees were recruited from a single National Cancer Institute–designated comprehensive cancer center. Initial recruitment emails were sent to physician-investigators known to be thoughtful about informed consent; snowball sampling was used to identify additional physician-investigator interviewees. Research managers from the cancer center identified a convenience sample of research coordinators, whom the study team invited via email. Patients were considered eligible if they were scheduled for an upcoming clinical appointment and had received a new cancer diagnosis at least 2 months prior. Research managers provided information about the study to eligible patients; contact information for interested patients was shared with the study team.

### Data Collection

Interview guides (eAppendix in Supplement 1) were developed by the authors (S.R.M., E.A.L., S.J.) and informed by a review of the literature.^2^ These guides explored respondents’ perceptions of and experiences with dual-role consent for adult oncology research, using probes to understand their views on role conflict, compromised voluntariness, and therapeutic misconceptions. Slight modifications were made for physician-investigator, research coordinator, and patient interviewees. Guides were pilot-tested and revised in response to feedback.

All interviews but 1 were conducted in English by a doctoral-level researcher (S.R.M. or D.B.); 1 patient interview was conducted in Spanish by a research coordinator using an institutional review board–approved translated interview guide. Interviews with physician-investigators were conducted in-person from July to September 2018. Interviews with research coordinators and patients were conducted via telephone or secure internet phone call between September 2020 and December 2022. Interviews were audio recorded and professionally transcribed. Interviews were a mean length of 22 minutes for physician-investigators, 50 minutes for research coordinators, and 21 minutes for patients. As diverse representation is a known challenge in oncology research,^29^ we collected self-reported data on race and ethnicity.

### Data Analysis

Analysis began during data collection and followed a thematic analysis approach^30^ to iteratively develop a codebook that included both theory-driven, a priori codes and emergent, inductive codes. Two authors (S.R.M., D.B.) independently reviewed a subset of transcripts to identify key themes and create a codebook. They double-coded all transcripts and met regularly to compare coding, rectify ambiguities, and refine the codebook; they subsequently drafted memos describing relevant themes and their frequency. All authors met to review and discuss these themes and their relationship to prior literature. Through review and discussion, the team concluded sufficient data had been collected to ensure theoretical saturation (the point at which the addition of new data does not alter the explanation of study findings).^31^

## Results

We conducted interviews with 43 respondents. Among the respondents, 28 (65.1%) were female; 9 (20.9%) were African American, 8 (18.6%) were Asian, 6 (14.0%) were Hispanic, and 21 (48.8%) were White; 15 were physician-investigators (6 [40.0%] with 6-10 years of experience, 4 [26.7%] with at least 20 years of experience), 13 were research coordinators (5 [38.5%] with 0-5 years of experience, 5 [38.5%] with 6-10 years of experience), and 15 were patients (9 [60.0%] aged 46-64 years) (eTables 1 and 2 in Supplement 1).

Four main themes were found. Interviewees (1) perceived greater potential for role synergy than for role conflict; (2) reported dual-role consent can have mixed effects on the consent process, increasing prospective participants’ understanding and likelihood of agreement while also threatening voluntariness; (3) preferred a team-based approach to the consent process; and (4) offered strategies for managing tensions in dual-role consent.

### Role Synergy, Not Role Conflict

Interviewees more often saw the roles of physician and investigator as complementary than as conflicting (Table 1). Nearly all physician-investigators and research coordinators spoke to physician-investigators’ subject matter expertise. As they described it, this expertise encompassed both (1) clinical knowledge, accumulated through medical education, specialized training, and professional experience, and (2) study-specific knowledge, including the nature of the study protocol, risks and potential benefits of participation, and differences between research and standard care. These interviewees expressed a belief that such subject matter expertise was beneficial for communicating with prospective patient-participants about research opportunities.

**Table 1.** Exemplary Quotes Regarding Role Synergy

Theme	Quote (participant)
**Physician-investigators’ subject matter expertise**	“I actually feel pretty strongly that [the research consent process should be led by a] medical oncologist, or whoever’s gonna be in charge of managing [the patient’s] treatment plan….[T]here are a lot of nuances with treatment that, really unless you deal with that on a sort of microscopic or daily level…, you really won’t be able to tell [prospective participants about] them in a great deal of detail. I mean, my practice often encounters dealing with cancer side effects or toxicities. You can only imagine trying to talk about the personal aspects of toxicities and side effects if you don’t really manage them.” (Physician-investigator 11)
	“There’s just no amount of clinical research experience that’s going to replace 4 years of med school, years of residency, and years of fellowship.…[I]f [prospective participants] started asking in-depth questions, sooner or later you’re going to find deficiencies in knowledge or gaps in knowledge that I don’t think it’s reasonable to ever expect a coordinator or a research nurse to be able to answer.” (Research coordinator 21)
	“[T]here’s so much happening in the treatment of various forms of cancer…when I’m talking to my oncologist, I hope I’m talking to somebody who is participating in developing the field and making the field progress. And if not, then I’m not sure if I’m getting up-to-date, current treatment [information].” (Patient 22)
**Physician-investigators’ patient-specific expertise**	“The treating physician probably knows the best about the patient, from the very beginning, the time it was diagnosed. I think they can sit down and actually go through what the disease is all about again.…[T]hey can set goals about the physiology of the disease, go through the standard of care treatments. Then actually describe more about where the clinical trial fits into that picture and exactly describe what the benefits and the risks are with that particular clinical trial.” (Physician-investigator 10)
	“It’s somebody that you’ve established a relationship with. You’ve discussed options at various different stages, and you’ve built up a relationship where you can discuss things in a way where perhaps you can slant your communication in a style that particularly fits that family because you know them well and they’ve got a relationship with you that hopefully involves them feeling comfortable asking different sorts of questions.” (Physician-investigator 7)
	“I think [my treating physician] understands me….[W]e all are different types of learners and some of us, just the way we comprehend things are different. So she understands how to talk to me. And she has an idea of what’s probably running through my head as she’s saying these things. And so I think I would just have a better understanding of what’s going on if it’s coming from her.” (Patient 14)

In addition to subject matter expertise, several physician-investigators mentioned the benefits of patient-specific expertise. This was understood as deep knowledge of the patient’s situation and values. Physician-investigators, they explained, have unique insights into the individual patient’s disease course and treatment regimen and can bring those insights to bear in discussions about study participation. Furthermore, relational rapport allows for the tailoring of communication styles to meet a patient’s needs and preferences.

Several interviewees acknowledged the potential for role conflict. They noted that a physician-investigator’s research-related interests may not always align with a patient’s best interests. Research-related interests included academic and scholarly incentives associated with trial accrual; financial interests were mentioned occasionally. While recognizing the possibility of role conflict, these interviewees characterized their concerns as manageable; management strategies are discussed later.

The strength of these benefits and concerns may depend on a physician-investigator’s role within the study in question. Physician-investigators and research coordinators occasionally drew distinctions based on whether the physician-investigator was a principal investigator (PI) or coinvestigator. This cut both ways. PIs were favorably described as having (1) greater familiarity with the details of the study, which made them likelier than coinvestigators to provide comprehensive information to prospective participants; and (2) greater investment than coinvestigators in the study’s success, which led them to more consistently offer participation to eligible individuals. Yet PIs also stand to benefit more from trial accrual, increasing the potential for role conflict.

Many patients expressed a preference to discuss trial participation with their treating physician. Views on the blending of the physician and investigator roles were, however, mixed. Some saw such blending in a positive light; for them, wearing 2 hats (physician and investigator) was a sign of expertise and stature in the field. Others expressed discomfort, sensing the potential for conflicts of interest.

### Impact on Consent

Interviewees felt dual-role consent could affect all aspects of informed consent: understanding, appreciation, and voluntariness. Physician-investigators and research coordinators valued physician-investigators’ subject matter expertise and patient-specific expertise. Previously, we highlighted how such expertise was understood to make the physician-investigator a uniquely valuable communicator of information; here, we focus on how the message may affect the receiver (ie, the prospective participant). Physician-investigators and research coordinators expressed a belief that physician-investigators could advance prospective participants’ understanding of a study by drawing on subject matter expertise to provide a robust description of the study and advance prospective participants’ appreciation by drawing on their knowledge of and relationship with the patient to contextualize what participation might mean for the individual in light of personal factors.

While seeing potential benefits, physician-investigators and research coordinators also noted potential challenges (Table 2). Dual-role consent might reduce therapeutic misconception if the physician-investigator could draw on their expertise to clearly articulate the research-care distinction, but it might alternatively promote therapeutic misconception if patient-participants misconstrued the physician-investigator’s role, continuing to see them solely as a physician, and so mistook research for medical care. Patient interviewees did not raise this concern.

**Table 2.** Exemplary Quotes Regarding Physician-Investigator Affect on Consent

Theme	Quote (participant)
**Therapeutic misconception**	“[Distinguishing research from care]…probably is going to be a conversation [with] some physician, because you really need to have a good understanding of ‘compared to what?’….[A research coordinator] may do a very good job of saying ‘This is what’s standard in the trial and this is what’s experimental in the trial,’ but he or she probably then is going to be only understanding all of it in the context of the trial itself.…[H]e or she probably doesn’t have the experience with a lot of patients on that standard of care.” (Physician-investigator 3)
	“I think certainly if a treating physician seems to be very excited or speak highly of a trial that may be misconstrued [by] patients.” (Physician-investigator 9)
**Voluntariness**	“If you’re the [principal investigator] of the trial, you want that trial to enroll, because that’s always the hardest thing on clinical trials. You’re inherently biased to get the patient to want to enroll and it’s very important to you. I think human nature being what it is, that can bleed through into your consent process.” (Physician-investigator 1)
	“[S]ometimes patients feel like they don’t want to disappoint their provider. I don’t think that they think that they’re doing it [agreeing to research participation] because they think that, ‘Oh, the oncologist or the surgeon is going to be mad at me.’ But I think that they’re like, ‘This person cares about me, and I don’t want to disappoint them.’ Like a kid doesn’t want to disappoint their parents, or something.” (Research coordinator 24)
	“I think it would make it a little bit more difficult [to decline participation if the treating physician is also an investigator in the study]. I mean, for me, if that’s the way I felt, I would say no. I would, but I probably might feel a little awkward about it….If the physician is also an investigator, it really is important for them to distinguish the option versus therapy.” (Patient 2)
**Receptivity to clinical trial participation when treating physician is involved in consent process**	“Especially cancer patients who stick with their oncologist for many years together and get one line of treatment after another, there’s a trust and a bond that the patient will look fondly upon if the treating physician offers [research participation] versus someone from outside.” (Physician-investigator 6)
	“I can tell a patient the exact same thing that the physician will say but they don’t believe me until the physician says it.…I just feel like that sense of trust that needs to be established….Because when patients get manipulated by so many people in the system they tend to look for that lighthouse, right, that person. And they’re like, well, he’s a physician. He went to school for umpteen years. He’s got MD behind his name. He’s the only person I’m going to listen to.” (Research coordinator 10)
	“I’ve been on a study that [my treating physician] recommended and I trust him implicitly. And I’m very aware that the study, he was there to advance science, but I trusted that he had my back. He wouldn’t put me on something that wasn’t good for me that was potentially going to be dangerous, et cetera. So, and it gave me the peace of mind. That’s why I would want to hear…from him about a study.” (Patient 3)

Physician-investigators and research coordinators acknowledged that dual-role consent might undermine the voluntariness of consent. These interviewees spoke to the inherent asymmetry of the physician-patient relationship, characterized by patients’ dependence on their physicians. They expressed concern that this power differential might cause patients to fear that declining research participation would undermine the preexisting therapeutic relationship. Patients, they noted, do not want to disappoint their physicians. Patients acknowledged the potential awkwardness of declining to participate in a study of which their physician was part. Nevertheless, most felt this awkwardness would not compromise their own decision-making, stating that they would be comfortable declining research participation.

Interviewees from all 3 groups reported that dual-role consent increased the likelihood of enrollment in research. Some noted that the involvement of the treating physician might increase a prospective participant’s receptivity to research-related discussions. They attributed this receptivity to factors such as patients’ familiarity with and trust in their physicians, which they felt the high-stakes nature of cancer care might heighten, and also to the expectation that physicians act in patients’ best interests. Some noted that increased enrollment might, problematically, reflect compromised voluntariness.

### Preference for a Team-Based Approach

All but 1 physician-investigator and all research coordinators preferred a team-based approach to consent (Table 3). They felt a physician-investigator should introduce the trial, discussing relevant scientific and clinical factors, before handing the process off to a research coordinator. Notably, their current practice generally reflected this approach. Few patients expressed an explicit preference for a team-based approach, but many emphasized a range of desires, including involvement of individuals with relevant expertise and presentation of information in a lay-friendly manner, consistent with what physician-investigators and research coordinators understood as the strengths of a collaborative approach.

**Table 3.** Exemplary Quotes Regarding Preference for a Team-Based Approach to Informed Consent

Theme	Quote (participant)
**Preference for team-based approach**	“How do you avoid those [conflicts of interest]? Well I think this is why the research coordinator is important....I don’t think there’s any way to completely disassociate [the physician-investigator from the conflict] or to resolve that conflict of interest. It’s gonna be there for a lot of things that we do…But a lot of that has to do with the individual and the conversations and the system it’s set up—we try to minimize it.” (Physician-investigator 2)
	“I think it’s good when [physicians] introduce [the study], they explain it to the patient as well. So when I go in and the patient may have medical questions, I can’t answer those. So that time to me, when a doctor introduces the study to the patient, it’s a perfect time to allow the patient to ask the doctor who has the best answers, those questions that they need to feel comfortable with the study. I believe that coordinators should only go in there to let the patient know their rights, to confirm that they understood what they were doing. And also if like there’s certain studies like, ‘Hey, we have to get a CT scan every 9 weeks.’…then that I can understand for the coordinators…to explain [that].” (Research coordinator 3)
**Role of research coordinator in promoting patient understanding**	“[Research coordinators] probably are going to know the nitty-gritty of that trial better than I do. You know, they have absorbed the protocol in ways that I never will.…[W]hat life is going to be like and when you’re really going to have to be back and forth and in the hospital or paying for what or whatever the case may be, they’re going to know that a lot better [than me]. I think that’s probably just as important for a lot of patients as how well the therapy’s going to work…[T]he coordinators know all of the aspects of the trial, what to expect in a lot more granular detail, so I think to be able to provide that perspective is going to be very helpful. So ‘this is really what you’re getting into,’ as opposed to conceptually ‘what you might experience.’” (Physician-investigator 3)
	“…[T]here’s a lot of pressure on us now as clinicians to produce RVUs and it takes a lot of time to do a proper consent…there may be issues with a busy clinic, and turning over that room to get the next patient in, and expectations about productivity that would maybe influence that clinician to abbreviate the consent process…because they have other pressures and other issues. Whereas, if you pull that patient out of the clinic room…and let a third party do the consent it gets rid of that problem.” (Physician-investigator 1)
**Role of research coordinator in promoting voluntariness**	“If it’s the research coordinator or another member of the research team…[prospective participants] feel more comfortable asking the questions or raising the concerns…than they are to ask [those questions of] their physician…I also think they view the person introducing the study, which is the treating physician, as kind of an interested party…so it is easier to say no to a person who works for [the treating physician]…than to say no to that person themselves.” (Physician-investigator 7)
	“[Research coordinators] have the time to discuss and to go into detail….But I think primarily [their role] is to avoid any bias, and the feeling of coercion by the patient.” (Research coordinator 20)

Abbreviation: CT, computerized tomography.

Both physician-investigators and research coordinators described a critical role for research coordinators in the consent process. There was general consensus that research coordinators could make distinct contributions to participant understanding. Coordinators, they explained, have more granular knowledge than physician-investigators of research-related logistics, such as the length and frequency of research visits as well as associated costs, and can offer prospective participants a clearer picture of the demands of participation in advance of a decision to participate. Furthermore, research coordinators experience fewer time pressures and so have more time for discussion. In addition, they are often better at using lay-friendly language. The involvement of research coordinators may also promote voluntariness; prospective participants may be more comfortable saying no to coordinators than to physician-investigators.

Views were mixed on whether research coordinators could address therapeutic misconceptions. Several physician-investigators volunteered that they deliberately inform prospective participants about the research-care distinction but still articulated a role for research coordinators in reinforcing the difference between research and care. Other physician-investigators stated that, because of the depth of knowledge of both research and care required, it is unreasonable to expect research coordinators to address the research-care distinction.

### Managing Dual-Role Consent

Interviewees offered a range of strategies to mitigate concerns about dual-role consent, including (1) repeatedly emphasizing to prospective participants that participation in research is voluntary and that refusal to participate will not negatively affect the treatment relationship; (2) presenting research participation as one among a range of options, including standard-of-care therapies, rather than as a yes-no decision; (3) disclosing conflicts of interest in the consent process; (4) providing ample time for the consent process to unfold, including time for prospective participants to talk with trusted others; and (5) where permitted by law,^32^ having a research coordinator, rather than a physician-investigator, obtain signatures on consent form (Table 4). All interviewees except 1 were opposed to prohibiting dual-role consent. This opposition reflected the perceived importance of physician-investigator’s expertise as well as patients’ desire to discuss research participation with their treating physician. The 1 exception, a surgeon-investigator, felt the power imbalance between the surgeon and prospective participant was too substantial a threat to voluntariness to allow dual-role consent.

**Table 4.** Strategies for Managing Dual-Role Consent

Theme	Quote (participant)
**Emphasize the voluntariness of participation**	“We stress [voluntariness] a lot during our consent process. I think several times letting [prospective participants] know that this is definitely voluntary, that they are able to withdraw any time that they would like, and nothing would change their standard of care with their physician. We stress that a lot just to let them know that they are able to withdraw…whenever they want.” (Research coordinator 11)
**Present trial as one among several options**	“I think that the ideal situation is for a patient to sort of have a menu of different options and to pick from among them and to have it laid out that way to say, ‘Look, you could do this standard of care or this trial, this other trial. Here’s the information. What do you think? What do you want to do?’ As opposed to saying, ‘Look, here’s a trial. Do you want to go on it and if you don’t, then we’ll talk about something else.’ I don’t know the answer to that, ‘cause I do tend to do the menu and not the true-false or the yes-no, right?” (Physician-investigator 3)
**Disclose conflicts of interest during the consent process**	“I guess the other thing that should be disclosed I think is if there’s any financial interest going on between the doctor and the company, the organization has conducted the study.” (Patient 3)
**Provide ample time for the consent process**	“I think the risk of coercion is always there, and I’ve seen it. But I think by giving some time for the patient to think about it, and the physician to back themselves out of that sense of urgency, that becomes less. I almost never ask somebody to consent the same day that I give them the idea of the trial….I usually let them go home. That allows them the chance to think about it, number 1....Also, people will frequently come in and say, ‘Well, I’d like to participate, but my mom won’t let me.’ Or, ‘My husband won’t let me.’ Or, ‘My wife won’t let me.’ Or, ‘My kids don’t think it’s a good idea.’ That gives them the chance to use that as an out.” (Physician-investigator 5)
**Have research coordinators obtain signatures on form**	“Getting their signature on something [for research] should be from somebody who is separate from their whole other standard of care. I think the division of standard of care and research is necessary. That’s why I think the research coordinator should consent the patient. It’s our job to do so. Again, to eliminate any feelings of the patient, feeling like they’re influenced or coerced into the study, or they think it’s part of their standard of care or their treatment.” (Research coordinator 17)

## Discussion

Guidelines caution against dual-role consent, reflecting concerns that it can create role conflict for physician-investigators and that prospective patient-participants may experience therapeutic misconceptions or feel the voluntariness of their decisions is compromised. However, current practices in adult oncology research often contravene this guidance. We interviewed physician-investigators, research coordinators, and patients to better understand their views on dual-role consent and identify a path forward.

Interviewees generally supported dual-role consent. While not ignoring potential challenges, their responses highlighted numerous ways in which dual-role consent might advance informed consent, particularly by promoting prospective participants’ understanding and appreciation. Dual-role consent also appeared to be consistent with patient preferences, as patients described a strong preference for involving their treating physicians in decisions about research. While this preference is not in and of itself decisive, it nevertheless merits particular attention, given informed consent is an instantiation of respect for persons.^33,34^

Considered together, our findings provide support for a hybrid or team-based approach to informed consent, involving both physician-investigators and third parties, such as research coordinators.^35,36^ Our data suggest physician-investigators and research coordinators share responsibilities to ensure that prospective participants’ adequately understand and voluntarily participate in research. Physician-investigators may be best positioned to provide clinical and scientific context, which can aid patient-participants in distinguishing research from care; research coordinators may be better able to describe the nitty-gritty of trial participation. While research coordinators appear to play a critical role in ensuring that participants feel free to decline research participation without fear of clinical repercussions,^35^ physician-investigators are responsible for reinforcing this message.

Notably, interviewees articulated an order of operations or sequence in which team members should act. They overwhelmingly preferred that physician-investigators initiate discussions about research participation. Only after the physician-investigator opened the door should the research coordinator enter the conversation. This reflected interviewees’ sense that a prospective participant’s familiarity with and trust in the physician-investigator increases comfort and willingness to discuss research. This finding is consistent with prior pediatric oncology studies that have described familiarity and trust as supportive of prospective participants’ openness to considering research participation; conversely, patients and families may resent being cold called by a team member with whom they have no prior relationship.^17,20,37^

Collectively, our findings support the argument that strict prohibition of dual-role consent is inadvisable. Dual-role consent might actually promote, rather than undermine, some goals of consent.^2,33,36^ Context, however, matters. As we and others have previously argued, the impact and value of dual-role consent may vary with features of trial design or patient demographics.^2,34,38^ It may also, as suggested by our interviewees, vary with the physician-investigator’s degree of study involvement.^3^ Physician-investigators and research coordinators perceived both the advantages and disadvantages of dual-role consent to be higher when the physician-investigator seeking consent is a PI rather than a coinvestigator. Although PIs typically have greater knowledge of the study to share with participants, their greater responsibility for the study’s success might create conflicts of interest, biasing how they portray the study to prospective patient-participants.

Finally, several of the mitigation strategies proposed herein are consistent with earlier recommendations from patient-participants or their families: allow adequate time for decision-making, involve family or trusted others in discussions, and present research as 1 of several options.^39^ Future empirical research could inform decisions about how best to implement these into a team-based approach and capitalize on the potentially advantageous features of dual-role consent while minimizing its risks. This is particularly urgent given the rise of precision oncology and the use of research as a means to access new diagnostics and therapeutics.^40^

### Limitations

This study had limitations. Our study focused on adult oncology research within a single comprehensive cancer center with a robust portfolio of industry- and government-funded oncology research. The majority of patients interviewed had previously participated in a clinical trial and held their treating physicians in high regard. The responses elicited in this context may not reflect the views of patients more broadly.

## Conclusion

This qualitative study explored the challenges and advantages of dual-role consent from the perspectives of key stakeholders, and the findings suggest that the advantages of dual-role consent in oncology may often outweigh the risks. Ultimately, our data offer support for a hybrid approach to informed consent, in which both physician-investigators and research coordinators collaborate and draw on their respective strengths to enhance the consent process for prospective participants. Further studies should test strategies to capitalize on the potential benefits of dual-role consent, such as role synergy and improved comprehension and appreciation, while addressing concerns, particularly around therapeutic misconception and the voluntariness of participants’ decision-making.

## Notes

Supplement 1. **eAppendix.** Interview Guides **eTable 1.** Physician-Investigator and Research Coordinator Characteristics **eTable 2.** Patient Characteristics (N=15)

Supplement 2.
Data Sharing Statement
