# Supplementary material for: Physician-Investigator, Research Coordinator, and Patient Perspectives on Dual-Role Consent in Oncology: A Qualitative Study
Source: JAMA Netw Open. 2023 Jul 25;6(7):e2325477. doi: 10.1001/jamanetworkopen.2023.25477 (PMC10369198; doi:10.1001/jamanetworkopen.2023.25477)
Supplement: Supplement 1. — eAppendix. Interview Guides eTable 1. Physician-Investigator and Research Coordinator Characteristics eTable 2. Patient Characteristics (N=15) [file jamanetwopen-e2325477-s001.pdf]

Supplemental Online Content

Morain SR, Barlevy D, Joffe S, Largent EA. Physician-investigator, research coordinator, and patient perspectives on dual-role consent in oncology: a qualitative study. *JAMA Netw Open.* 2023;6(7):e2325477. doi:10.1001/jamanetworkopen.2023.25477

eAppendix. Interview Guides

eTable 1. Physician-Investigator and Research Coordinator Characteristics

eTable 2. Patient Characteristics (N=15)

This supplemental material has been provided by the authors to give readers additional information about their work.

© 2023 Morain SR et al. *JAMA Network Open.*

eAppendix. Interview Guides

Who Should Invite Patients into Oncology Research?
Physician Interview Guide

Thank you again for agreeing to share your views with us about this important topic. As a reminder, this interview is part of a research study about the informed consent process for clinical trials in oncology. Our interview will last between 20-30 minutes.

With your permission, I would like to be able to audiorecord this interview. The recording and transcripts will be de-identified, and will not be shared with any individual beyond the members of our study team. We will not include your name in any publication or presentation resulting from this study. Would it be alright with you if we audiorecord this interview? *(confirm permission to audiorecord)*

1. **To start, I'd like to ask you to imagine that you have a patient who is eligible for a clinical trial for a new cancer therapy. Who, in your view, should be the person who discusses the study with your patient and asks whether he/she would like to participate?**

    - *If necessary, offer the option—yourself, or someone else?*
    - *Probe—why?*

2. **Now imagine that your hospital created a policy that would require that *you,* as the treating oncologist, should be the individual who informs your patient about a trial and asks whether he/she would like to participate. How would you think about such a policy?**

    - How might you expect this to impact your patients' understanding of a trial, including how it differs from what they would experience in clinical care?
    - How might you expect this to impact your patients' perception of the voluntariness of trial participation? Would patients be more or less willing to refuse if they didn't want to participate, if you were the one discussing the trial with them?
    - Would this create an issue of role conflict for you? In other words, would you feel your obligations as a researcher would demand something different from you from those obligations as a physician?

© 2023 Morain SR et al. *JAMA Network Open.*

3. **Now imagine that your hospital instead created a policy that would require that someone else NOT involved in the patient's care be the person who told patients about the trial and asked whether they would like to participate. How would you think about such a policy?**
4. **What have I not asked that we should be considering?**

5. **Who else to speak with?**

6. **Interviewing patients?**

© 2023 Morain SR et al. *JAMA Network Open.*

Who Should Invite Patients into Oncology Research?
Research Coordinator Interview Guide

Thank you again for agreeing to share your views with us about this important topic. As a reminder, this interview is part of a research study about the informed consent process for clinical trials in oncology. Our interview will last between 30-40 minutes.

With your permission, I would like to be able to audiorecord this interview. The recording and transcripts will be de-identified, and will not be shared with any individual beyond the members of our study team. We will not include your name in any publication or presentation resulting from this study. Would it be alright with you if we audiorecord this interview? *(confirm permission to audiorecord)*

1. **To start, we'd like to learn a bit about your experience with cancer clinical trials.**
    - How long have you been a research coordinator?
    - How many trials have you been a part of?
    - What has been the clinical focus of those trials?
    - What study phase(s)?

2. **Thinking about the trial(s) in which you've been involved, can you describe to me what the recruitment and consent process generally looks like? Specifically, I'm interested in knowing how patients first learn of trials, and who discusses participation with them.**
    - What role do trial investigators typically play in recruitment and consent?
    - What role do the primary/treating oncologists typically play, if they are not themselves trial investigators?
    - What role do research coordinators typically play?
    - How, if at all, is the consent process different if the treating physician is ALSO one of the investigators for the trial?
    - Do other professionals typically play any role in these processes? (e.g. research nurses)
    - Do these roles typically vary by type of trial (e.g., Phase I, Phase II, Phase III)?
    - *Probe—if a division of labor, probe as to why*

3. **One more follow-up about the recruitment and consent processes with which you've been involved—what does the timeline look like? Specifically, how much time is there between recruitment and study enrollment?**

4. **How do you feel about the recruitment and consent process just described? How do you think it works for the team? For patients?**

© 2023 Morain SR et al. *JAMA Network Open.*

- *Probe, if needed: Have you ever had any questions or concerns about the recruitment and consent process? If so, what were they? Have you discussed these concerns with anyone? Who? What was their response?*

5. **I'd like to hear your thoughts about the role of treating oncologists in telling patients about a trial and seeking their informed consent, especially when that treating oncologists are also trial investigators.**
    - What do you think are the advantages of having a treating oncologist who is also a trial investigator tell their patient about a trial and/or seek their informed consent to participate?
    - What are the disadvantages?

6. **Now, I'd like to ask you to imagine that you have the opportunity to plan out the consent process for a new cancer clinical trial. Who, in your view, *should* be the person who discusses the study with a patient and asks whether he/she would like to participate?**
    - *If necessary, offer the option—the treating physician? The study PI? Yourself? Someone else? Should different people have different roles in the process?*
    - *Probe—why?*
    - *Who do you think is best positioned to help prospective trial participants <u>understand</u> the study, and how it differs from clinical care?*
        - *Are there some parts of the study that are better explained by different individuals? Which ones, and by whom?*
    - *Who do you think is best positioned to help ensure that prospective participants feel that it is truly <u>their choice</u> to participate—in other words, that the decision is fully voluntary?*
    - *(If answers to the above probes are different): How, in your view, can we best balance the goals of understanding and voluntariness?*

7. **Now imagine that your hospital created a policy that would require that the treating oncologist *must* be the individual who informs a patient about a trial and asks whether he/she would like to participate. How would you think about such a policy?**
    - How might you expect this to impact patients' understanding of a trial, including how it differs from what they would experience in clinical care?

- How might you expect this to impact patients' perception of the voluntariness of trial participation? Do you think patients might be more or less willing to refuse if they didn't want to participate, if their treating physician were the one discussing the trial with them?

- What about if the treating physician is also a trial investigator? Do you think this would have a different impact on patient understanding and/or voluntariness? Why/why not? How?

8. **Now imagine that your hospital instead created a policy that would require that the treating oncologist NOT be the person who told patients about the trial and asked whether they would like to participate. How would you think about such a policy?**

    - How might you expect this to impact patients' understanding of a trial, including how it differs from what they would experience in clinical care?

    - How might you expect this to impact patients' perception of the voluntariness of trial participation? Do you think patients might be more or less willing to refuse if they didn't want to participate, if their treating physician were not the one discussing the trial with them?

9. **Are there any additional issues related to research informed consent that should be considered when the treating oncologist is the study's principal investigator?**

10. **What have I not asked that we should be considering? Is there anything else you'd like to add?**

11. **Who else should we speak with about this issue?**

© 2023 Morain SR et al. *JAMA Network Open.*

Who Should Invite Patients into Oncology Research?
Patient Interview Guide

Thank you again for agreeing to share your views with us about this important topic. As a reminder, this interview is part of a research study about the informed consent process for clinical trials in oncology. Our interview will last between 20-30 minutes.

With your permission, I would like to be able to audiorecord this interview. The recording and transcripts will be de-identified, and will not be shared with any individual beyond the members of our study team. We will not include your name in any publication or presentation resulting from this study. Would it be alright with you if we audiorecord this interview? *(confirm permission to audiorecord)*

1. **To start, could you tell me if you have participated or are currently participating in a clinical trial for cancer?**

    - *If yes—could you tell me a bit about that trial? What was the purpose of the study?*
    - *Please describe, to the extent that you remember, how you first learned about the trial and how you decided to participate?*
    - *Probes:*
        - i. *Who introduced the trial?*
        - ii. *Who described what participation would entail?*
        - iii. *What was the time between learning of the trial and making a decision to participate?*
        - iv. *When and with whom did you sign the consent form?*
    - *Did you have any concerns about that process? Is there anything you think could have been done differently to help you make the decision?*

2. **I'd like to ask you to imagine that you are eligible for a clinical trial for a new cancer therapy. For this trial, like all clinical trials, you would first go through an informed consent process, in which you were given information about the study and the potential risks and benefits of participation. Who, in your view, should be the person who discusses the study with you and asks whether you'd like to participate?**

    - *If necessary, offer the option—your oncologist, or someone else?*
    - *Probe—why do you prefer that person discuss the trial with you?*

© 2023 Morain SR et al. *JAMA Network Open.*

3. **Now imagine that the hospital where this trial was to take place had created a policy that would require that your oncologist was the person who had to tell you about the trial and ask whether you would like to participate. How would you think about such a policy?**

    - One of the things we want to make sure of is that patients have a *choice* whether or not to participate in studies. Would you be comfortable saying "no", you didn't want to participate, if your physician was the one discussing the trial with you?

    - How would you think about your physician being both the person who provides your health care, but also is involved in research studies like this one?

4. **Now imagine that the hospital where this trial was to take place had created a policy that would require that your oncologist was NOT the person who had to tell you about the trial and ask whether you would like to participate. Instead, a person who was not involved in your clinical care would be the person who would discuss the trial with you. How would you think about such a policy?**
    - In which of the two approaches would you feel you were able to better understand the study—if you discussed it with your own physician, or with a person who was not involved in your clinical care?

What didn't I ask you about that I should have? What else is important for us to think about related to informed consent for cancer studies?

© 2023 Morain SR et al. *JAMA Network Open.*

eTable 1. Physician-Investigator and Research Coordinator Characteristics

	Physician-Investigators, n=15	Research Coordinators, n=13
**Sex, No. (%)**		
Female	6 (40.0)	10 (76.9)
Male	9 (60.0)	3 (23.1)
**Race/Ethnicity, No. (%)**[1]		
African-American (Non-Hispanic)	1 (6.7)	4 (30.8)
Asian	6 (40.0)	1 (7.7)
Hispanic	1 (6.7)	4 (30.2)
White (Non-Hispanic)	7 (46.7)	5 (38.5)
**Specialization, No. (%)**		
Hematology & Oncology	14 (93.3)	NA
Surgery	1 (6.7)	NA
**Years of Experience**		
0-5	2 (13.3)	5 (38.5)
6-10	6 (40.0)	5 (38.5)
11-15	3 (20.0)	2 (15.4)
≥20	4 (26.7)	1 (7.7)

© 2023 Morain SR et al. *JAMA Network Open.*

1. Individuals may identify as more than one race/ethnicity

eTable 2. Patient Characteristics (N=15)

**Sex, No. (%)**	
Female	12 (80.0)
Male	3 (20.0)
**Race/Ethnicity[1]**	
African-American (Non-Hispanic)	4 (26.7)
Asian	1 (6.7)
Hispanic	1 (6.7)
White (Non-Hispanic)	9 (60.0)
**Age, No. (%), y**	
46-64	9 (60.0)
≥65	6 (40.0)
**Highest Level of Education**	
High School	3 (20.0)

© 2023 Morain SR et al. *JAMA Network Open.*

Some College	3 (20.0)
Bachelor's Degree	2 (13.3)
Graduate Degree	7 (46.7)
**Diagnosis, No. (%)**	
Breast Cancer	10 (66.7)
Colon Cancer	1 (6.7)
Pancreatic Cancer	4 (26.7)
**Prior Research Experience, No. (%)[2]**	
Drug (Phase I/II)	8 (53.3)
Drug (Phase III)	1 (6.7)
Device/Interventional	7 (46.7)
Tissue Banking	5 (33.3)
No Prior Research Experience	1 (6.7)

1. Individuals may identify as more than one race/ethnicity
2. Patient interviewees may have had experience in more than one prior research study.

© 2023 Morain SR et al. *JAMA Network Open.*
