# Supplementary material for: Physician-Investigator, Research Coordinator, and Patient Perspectives on Dual-Role Consent in Oncology: A Qualitative Study
Source: JAMA Netw Open. 2023 Jul 25;6(7):e2325477. doi: 10.1001/jamanetworkopen.2023.25477 (PMC10369198; doi:10.1001/jamanetworkopen.2023.25477)
Supplement: Supplement 2. — Data Sharing Statement [file jamanetwopen-e2325477-s002.pdf]

Data Sharing Statement

Morain. Physician-Investigator, Research Coordinator, and Patient Perspectives on Dual-Role Consent in Oncology. *JAMA Netw Open*. Published July 25, 2023.
doi:10.1001/jamanetworkopen.2023.25477

Data
Data available: No

Additional Information
Explanation for why data not available: The nature of qualitative data raises distinct considerations for privacy and confidentiality, particularly related to the potential for deductive disclosure. Furthermore, our initial consent promised participants that data would not be shared outside members of the study team.
